# Cerebrospinal fluid biomarkers profile in scans without evidence of dopaminergic deficits (SWEDD)

**DOI:** 10.1016/j.ibneur.2023.10.001

**Published:** 2023-10-20

**Authors:** Fardin Nabizadeh, Sara KamaliZonouzi, Maryam Noori

**Affiliations:** a School of Medicine, Iran University of Medical Sciences, Tehran, Iran; b School of Medicine, Tehran University of Medical Science, Tehran, Iran; c Student Research Committee, School of Medicine, Iran University of Medical Sciences, Tehran, Iran; d Urology Research Center, Tehran University of Medical Science, Tehran, Iran

**Keywords:** Parkinson’s disease, Scans without evidence of dopaminergic deficits, SWEDD, Cerebrospinal fluid (CSF), Alpha-synuclein, Tau, Beta-amyloid

## Abstract

**Background:**

A small proportion of patients with clinical parkinsonism have normal transporter-single photon emission computed tomography (DaTSPECT) which is commonly defined as scans without evidence of dopaminergic deficits (SWEDD). A better understanding of SWEDD can improve the current therapeutic options and appropriate disease monitoring.

**Aim:**

We aimed to assess CSF biomarkers levels including α-synuclein (α-syn), amyloid βeta (Aβ1–42), total tau (t-tau), and phosphorylated tau (p-tau) in SWEDD and investigate the longitudinal alteration in the CSF profile.

**Methods:**

In total, 406 early-stage PD, 58 SWEDD, and 187 healthy controls (HCs) were entered into our study from PPMI. We compared the level of CSF biomarkers at baseline, six months, one year, and two years. Furthermore, the longitudinal alteration of CSF biomarkers was explored in each group using linear mixed models.

**Results:**

There was no significant difference in the level of CSF α-syn Aβ1–42, t-tau, and p-tau between HCs and SWEDD at different time points. Investigating the level of CSF α-syn in PD and SWEDD showed a significant difference at one (*p* = 0.016) and two years (*p* = 0.006). Also, we observed a significant difference in the level of CSF Aβ1–42 between SWEDD and PD at one year (*p* = 0.012). Moreover, there was a significant difference in the level of CSF t-tau between SWEDD and PD subjects at one (*p* = 0.013) and two years (*p* = 0.017). Furthermore, there was a significant difference in the level of CSF p-tau between SWEDD and PD groups at two years visits (*p* = 0.030). Longitudinal analysis showed a significant decrease after one (*p* = 0.029) and two years (*p* = 0.002) from baseline in the level of CSF α-syn only in the PD group. Also, we observed that the level of CSF Aβ1–42 significantly increased after one year in SWEDD (*p* = 0.031) and decreased after two years from baseline in PD subjects (*p* = 0.005). Moreover, there was a significant increase in the level of CSF t-tau after two years (*p* = 0.036) and CSF p-tau after six months from baseline in SWEDD subjects (*p* = 0.011).

**Conclusion:**

This finding suggests a faster neurodegeneration process in PD patients compared to SWEDD at least based on these biomarkers. Future studies with longer follow-up duration and more sample sizes are necessary to validate our results.

## Introduction

Globally, Parkinson’s disease (PD) is the second leading cause of disability among neurodegenerative disorders in older adults (Vos et al., 2020). During the past decades, the prevalence of PD has doubled (Dorsey et al., 2018) and it is anticipated to rise in years to come following the rapid global transition to population aging (Rossi et al., 2018). PD is biologically characterized by losses of dopaminergic neurons in the brainstem particularly those in the substantia nigra (de Celis Alonso et al., 2015). The affected individuals mostly presented with resting tremors, muscular rigidity, bradykinesia, and postural instability (Poewe et al., 2017). In addition to PD, several nosologic entities that are grouped by their common clinical characteristics but differentiated by their unique pathologies are also included under the overarching term "parkinsonism”.

Most recently, dopamine transporter-single photon emission computed tomography (DaTSPECT) has been widely utilized for identifying the degeneration of presynaptic dopamine receptors in the nigrostriatal system (Ba and Martin, 2015). By this precise method of scanning, only a small proportion of patients with normal DaTSPECT are referred to as having atypical parkinsonism which is commonly defined as scans without evidence of dopaminergic deficits (SWEDD) (Schneider et al., 2007). In the initial course of the disease, SWEDD can be clinically diagnosed as PD; however, the subsequent functional imaging shows no evidence of presynaptic dopaminergic dysfunction (Erro et al., 2016). The exact etiology of SWEDD has not been completely understood and it was revealed that the frequency of some comorbidities such as cardiovascular disorders, orthostatic hypotension, and sleep disturbances are higher in patients suffering from SWEDD compared to PD (Nalls et al., 2015, Taylor et al., 2016). As a result, finding potential biomarkers that will differentiate the diagnosis of SWEDD from PD at the first steps of the disease progression may become in parallel with better management and improved overall survival of affected patients.

Cerebrospinal fluid (CSF) is a favorable source of CNS biomarkers for neurodegenerative disorders. The CSF α-synuclein (α-syn), amyloid βeta (Aβ)1–42, total tau (t-tau), and phosphorylated tau (p-tau) have been accounted as diagnostic, prognostic, and predictive biomarkers for PD (Parnetti et al., 2019). According to the results of several studies, the CSF total α-syn is lower in PD patients compared to the controls (Sako et al., 2014, Gao et al., 2015, Zhou et al., 2015, Eusebi et al., 2017). In terms of Aβ1–42, a consensus has not been reached and while a trend of lower CSF Aβ1–42 has been found in some investigations (Kang et al., 2013, Campbell et al., 2015, Stav et al., 2015, Delgado‐Alvarado et al., 2016), it was not confirmed by the others (Parnetti et al., 2014, Hall et al., 2015, Kang et al., 2016). In addition, a morbid complication of PD, known as Parkinson’s disease dementia (PDD) was associated with a higher level of CSF Aβ1–42 which is in accordance with the observed pattern for Alzheimer's disease (Nutu et al., 2013, Vranová et al., 2016). Likewise, studies on CSF t-tau and p-tau have not shown a specific profile for PD, with results of both lower and equal levels in PD compared to controls and other parkinsonian disorders (Kang et al., 2013, Parnetti et al., 2014, Parnetti et al., 2014, Hall et al., 2015, Magdalinou et al., 2015, Delgado‐Alvarado et al., 2016, Kang et al., 2016, Mollenhauer et al., 2017). Furthermore, Yu and colleagues performed a study to compare the CSF biomarkers of patients with PD and SWEDD with healthy controls. It was demonstrated that while PD was associated with decreased CSF α-syn, Aβ1–42, t-tau, and p-tau compared to controls, no difference was observed in the level of these biomarkers between PD and SWEDD patients (Yu and Li, 2021). However, to the best of our knowledge, there is no study comparing the pattern of changes in the level of these biomarkers during the course of the disease in both patients with PD and SWEDD.

Consequently, we sought for investigating how the CSF levels of α-syn, Aβ1–42, t-tau, and p-tau varied and changed over the progression of PD and SWEDD in addition to investigating whether the level of differences in the biomarkers could be used for differentiating these two conditions.

## Materials and methods

### Participants

The participants were recruited from Parkinson’s Progression Markers Initiative (PPMI, http://www.ppmi-info.org/). The study was approved by the institutional review board of all participating sites. Written informed consent was obtained from all participants before study enrolment. The study was performed based on relevant guidelines and regulations. PD was diagnosed according to Movement Disorder Society (MDS) criteria (Postuma et al., 2015), and disease severity was evaluated by Movement Disorder Society-Unified Parkinson's Disease Rating Scale (MDS- UPDRS), and dopamine transporter deficit was detected by DAT scan. We excluded the subjects with any neurological and psychiatric disorders apart from PD. In total, 406 early-stage PD, 58 SWEDD, and 187 healthy controls (HCs) were entered into our study. The drug naïve PD patients at Hoehn and Yahr (H&Y) staging I or II were only included.

### Motor and non-motor assessments

All participants completed motor and non-motor tests at the baseline visit assessed by PPMI staff. The H&Y staging and MDS-UPDRS were used for motor assessments. The Montreal Cognitive Assessments (MoCA) were performed to measure cognitive status, Letter Number Sequencing (LNS), and semantic fluency to assess executive function and working memory. Also, Epworth Sleepiness Scale (ESS) was applied to evaluate the patient's sleepiness. The olfaction function measured by the University of Pennsylvania Smell Identification Test (UPSIT) and Rapid eye movement (REM) sleep behavior disorder (RBD) was evaluated using REM Sleep Behavior Disorder Screening Questionnaire (RBDSQ).

### CSF assessments

CSF measures were obtained from the PPMI database for each patient via the following process. After testing cell count, total protein level, and glucose level, the frozen samples which were collected via standard lumbar puncture method were sent to PPMI Biorepository Core Laboratories and then transferred to the University of Pennsylvania for measurement of CSF total α-syn, Aβ1–42, t-tau, and p-tau. A commercial enzyme-linked immunosorbent assay kit (Covance, Dedham, MA) was used to assess CSF total α-syn (Kang et al., 2016). Also, Elecsys electrochemiluminescence immunoassays on the Cobas e 601 platforms (Roche Diagnostics, described at http://ppmi-info.org; project ID: 125) were used to analyze the CSF Aβ1–42, t-tau, and p-tau. We obtained the level of the CSF biomarkers at four visits (baseline, 6 months, 1 year, and 2 years)(Table 1).

**Table 1:** Number of participants with available CSF biomarkers at each time point.

Variable	HCs (n = 187)	SWEDD (n = 58)	PD (n = 406)
Baseline	187	58	406
6 months	155	40	243
1 year	147	43	294
2 years	132	35	286

HCs, healthy controls; PD, Parkinson's disease; SWEDD, scan without evidence of dopaminergic deficit

### Statistical analysis

All statistical analyses were performed using SPSS version 22 (BM Corp., Armonk, NY, USA). The normality of the data was checked using the Kolmogorov-Smirnov test. The t-test and Mann–Whitney U-test were performed for comparing the parametric and non-parametric variables between every two groups. Furthermore, we used linear mixed models within each group to assess the longitudinal changes of CSF biomarkers. The model included time from baseline as a fixed effect factor, CSF biomarkers as a dependent variable and each subject as random effect. The Benjamini-Hochberg method was used to address the type I error due to the multiple comparisons. *P*-values < 0.05 were regarded as significant.

## Results

### Clinical and demographical characteristics

The clinical and demographical characteristics of the participants are detailed in Table 2. Comparison analysis showed that SWEDD and PD had worse MDS-UPDRS, RBD, and UPSIT scores compared to the HCs. Furthermore, the SWEDD group showed significantly lower scores in MoCA and LNS and higher scores in ESS compared to HCs. There was also a significant difference in MDS-UPDRS parts I and III, UPSIT, and ESS scores between PD and SWEDD individuals.

**Table 2:** Comparison of clinical and demographical characteristics between HCs and patients with PD at baseline.

Variable	HCs (n = 187)	SWEDD (n = 58)	PD (n = 406)	*Adjusted P*-value (HCs vs SWEDD)	*Adjusted P*-value (HCs vs PD)	*Adjusted P*-value (SWEDD vs PD)
Age, mean (SD), years*	61.3 (11.1)	60.6 (10.0)	61.9 (9.6)	0.76	0.63	0.50
Female/Male, No* *	68/119	20/38	177/229	0.67	0.72	0.47
Left-handed/Right-handed, No* *	23/164	Aug-50	36/370	0.75	**0.04**	0.12
Education, mean (SD), years*	16.0 (2.9)	15.1 (3.8)	15.6 (2.9)	0.07	0.17	0.42
Disease duration, mean (SD), months* *	_	9.8 (5.9)	10.4 (6.3)		0.21	0.53
Hoehn and Yahr stage, mean (SD)* *	_	1.4 (0.5)	1.5 (0.5)		0.95	0.16
MDS-UPDRS part I score, mean (SD)*	0.5 (1.0)	2.0 (2.4)	1.2 (1.5)	**0.00**	**0.00**	**0.00**
MDS-UPDRS part II score, mean (SD)*	0.4 (1.0)	5.6 (5.2)	5.9 (4.1)	**0.00**	**0.00**	0.69
MDS-UPDRS part III score, mean (SD)*	1.2 (2.2)	14.6 (9.7)	20.9 (8.9)	**0.00**	**0.00**	**0.00**
RBD score, mean (SD)* *	3.8 (2.3)	6.4 (3.1)	5.6 (2.9)	**0.01**	**0.00**	0.11
UPSIT score, mean (SD)*	34.1 (4.7)	30.8 (7.5)	22.3 (8.2)	**0.01**	**0.00**	**0.00**
MoCA score, mean (SD)*	28.2 (1.1)	26.9 (2.5)	27.1 (2.3)	**0.01**	0.40	0.69
ESS score, mean (SD)* *	5.6 (3.4)	8.4 (4.8)	5.7 (3.4)	0.12	0.66	**0.00**
LNS score, mean (SD)*	10.9 (2.5)	9.7 (2.5)	10.5 (2.6)	0.12	0.18	0.12

HCs, healthy controls; PD, Parkinson's disease; SWEDD, scan without evidence of dopaminergic deficit; MDS-UPDRS, Movement Disorder Society-Sponsored Revision of the Unified Parkinson's Disease Rating Scale; RBD, RBD; REM sleep behavior disorder; UPSIT, University of Pennsylvania Smell Identification Test; MoCA, Montreal Cognitive Assessments, ESS, Epworth Sleepiness Scale; LNS, Letter Number Sequencing

*t-test

* *Mann-Whitney U-test

### Comparison of CSF biomarkers

**Fig. 1:**
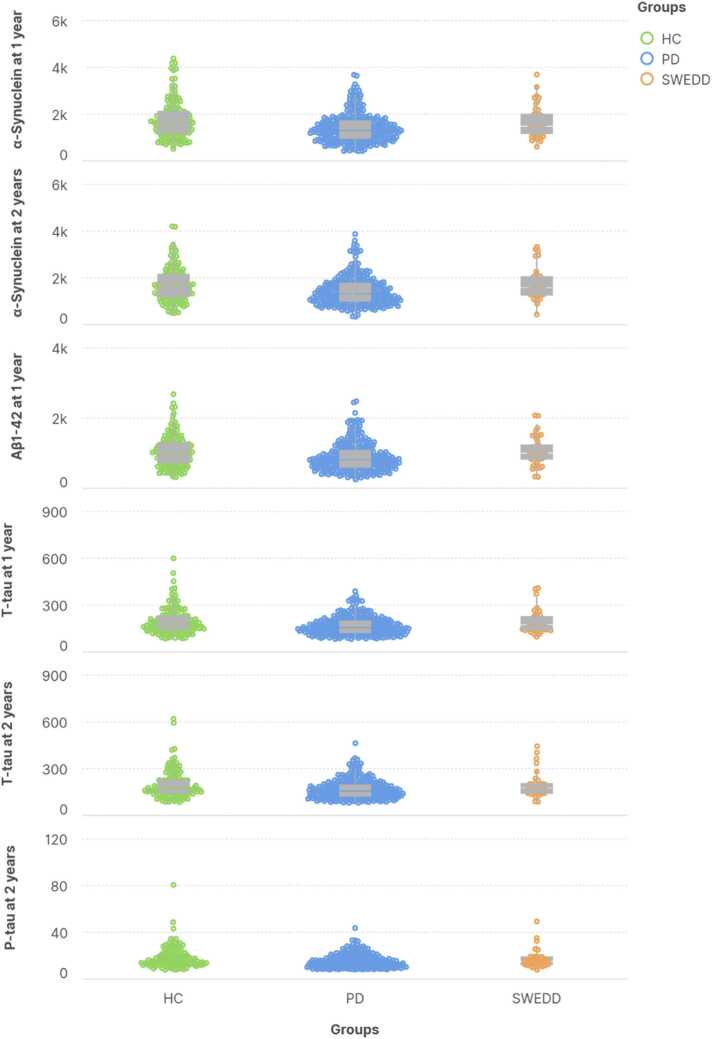
Significant results of comparisons between groups. The level of the CSF biomarkers in SWEDD (orange), PD (blue), and HC (green) groups in one year and two years from baseline.

There was no significant difference in the level of CSF α-syn Aβ1–42, t-tau, and p-tau between HCs and SWEDD at different time points (Table 3). Investigating the level of CSF α-syn in PD and SWEDD showed a significant difference at one (*p* = 0.016) and two years (*p* = 0.006) but not at baseline (*p* = 0.122) and six months (*p* = 0.746) (Fig. 1). We observed a significant difference in the level of CSF Aβ1–42 between SWEDD and PD at one year (*p* = 0.012) but not at other time points. Further analysis showed a significant difference in the level of CSF T-tau between SWEDD and PD subjects at one (*p* = 0.013) and two years (*p* = 0.017). Also, there was a significant difference in the level of CSF P-tau between SWEDD and PD groups at two years visits (*p* = 0.030).

**Table 3:** CSF biomarker comparisons and longitudinal changes.

Variable	HCs (n = 187)	SWEDD (n = 58)	PD (n = 406)	*P*-value (HCs vs SWEDD)	*P*-value (HCs vs PD)	*P*-value (SWEDD vs PD)
α-Synuclein						
Value at baseline, mean (SD), pg/ml*	1696.7 (754.2)	1660.3 (728.9)	1512.5 (671.8)	0.747	**0.003**	0.122
Value at 6 months, mean (SD), pg/ml*	1785.0 (767.6)	1571.0 (484.6)	1529.2 (793.0)	0.095	**0.002**	0.746
Value at 1 year, mean (SD), pg/ml*	1767.9 (796.4)	1658.4 (669.8)	1414.6 (605.7)	0.413	**< 0.001**	**0.016**
Value at 2 year, mean (SD), pg/ml*	1710.8 (723.3)	1738.2 (689.1)	1432.7 (609.1)	0.841	**< 0.001**	**0.006**
*P*-value within linear mixed model till 6 months	0.27	0.87	1.00			
*P*-value within linear mixed model till 1 year	0.27	0.48	**0.05**			
*P*-value within linear mixed model till 2 years	0.74	0.69	**0.02**			
Aβ1–42						
Value at baseline, mean (SD), pg/ml*	1018.1 (499.0)	956.0 (350.6)	910.8 (411.8)	0.38	**0.006**	0.427
Value at 6 months, mean (SD), pg/ml* *	1043.8 (456.6)	942.0 (261.6)	941.9 (412.9)	0.18	0.023	0.999
Value at 1 year, mean (SD), pg/ml*	1058.0 (456.2)	1053.9 (399.1)	889.0 (397.5)	0.96	**< 0.001**	**0.012**
Value at 2 year, mean (SD), pg/ml*	1049.3 (518.3)	1017.1 (350.9)	876.5 (380.3)	0.73	**< 0.001**	0.038
*P*-value within linear mixed model till 6 months	0.25	0.72	1.00			
*P*-value within linear mixed model till 1 year	0.33	**0.04**	0.80			
*P*-value within linear mixed model till 2 years	0.39	0.64	**0.03**			
T-tau						
Value at baseline, mean (SD), pg/ml*	192.1 (79.8)	177.3 (60.8)	169.7 (57.8)	0.20	**< 0.001**	0.356
Value at 6 months, mean (SD), pg/ml*	197.1 (81.4)	180.1 (58.6)	171.6 (62.5)	0.22	**0.001**	0.428
Value at 1 year, mean (SD), pg/ml*	197.6 (84.4)	190.7 (72.1)	166.3 (57.5)	0.63	**< 0.001**	**0.013**
Value at 2 year, mean (SD), pg/ml* *	199.8 (88.9)	195.4 (80.8)	168.0 (61.5)	0.79	**< 0.001**	**0.017**
*P*-value within linear mixed model till 6 months	0.29	0.14	1.00			
*P*-value within linear mixed model till 1 year	0.40	0.12	0.98			
*P*-value within linear mixed model till 2 years	0.26	**0.04**	1.00			
P-tau						
Value at baseline, mean (SD), pg/ml*	17.5 (8.4)	15.6 (5.9)	14.9 (5.3)	0.12	**< 0.001**	0.348
Value at 6 months, mean (SD), pg/ml*	17.6 (8.5)	16.0 (6.2)	15.1 (5.4)	0.29	**0.001**	0.322
Value at 1 year, mean (SD), pg/ml*	18.1 (9.1)	16.5 (7.1)	14.8 (5.2)	0.29	**< 0.001**	0.058
Value at 2 year, mean (SD), pg/ml*	18.1 (9.3)	17.1 (8.2)	14.8 (5.5)	0.57	**< 0.001**	**0.03**
*P*-value within linear mixed model till 6 months	0.63	**0.04**	1.00			
*P*-value within linear mixed model till 1 year	0.78	0.20	1.00			
*P*-value within linear mixed model till 2 years	0.25	0.15	0.98			

HCs, healthy controls; PD, Parkinson's disease; SWEDD, scan without evidence of dopaminergic deficit Longitudinal changes within the liner mixed model in each group at each time point and their changes from baseline.

Significant results are bolded

*t-test

* *Mann-Whitney U-test

### Longitudinal changes in CSF biomarkers

**Fig. 2:**
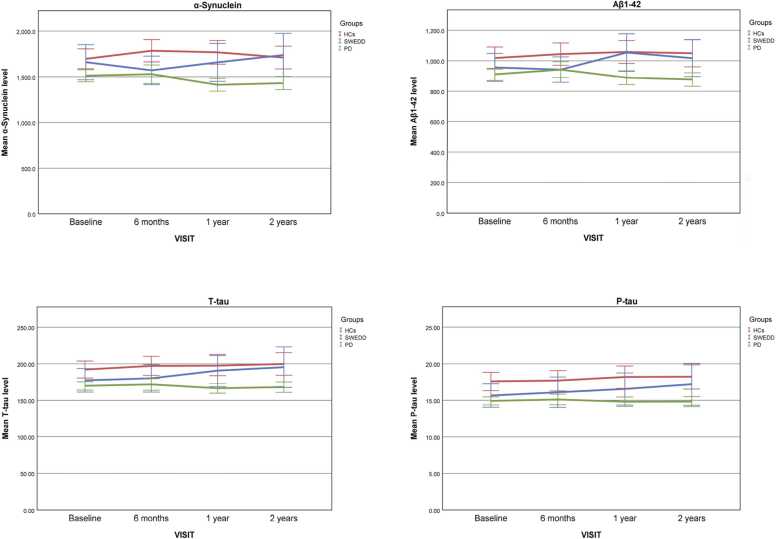
Representation of CSF α-syn, P-tau, T-tau, and Aβ1–42 levels at different timepoints for SWEDD (blue), PD (green), and HCs(red) groups. Error bars indicate 95% confidence interval.

The linear mixed model analysis showed significant changes after one (*p* = 0.029) and two years (*p* = 0.002) from baseline in the level of CSF α-syn only in the PD group not in SWEDD or HCs (Fig. 2). Also, we observed that the level of CSF Aβ1–42 significantly increased after one year in SWEDD (*p* = 0.031) and decreased after two years from baseline in PD subjects (*p* = 0.005). The CSF T-tau and P-tau levels did not change during follow-up in the HCs and PD group, however, we detected a significant increase in the level of CSF T-tau after two years (*p* = 0.036) and CSF P-tau after six months from baseline in SWEDD subjects (*p* = 0.011).

## Discussion

We conducted a cross-sectional study to investigate the levels of CSF biomarkers including α-syn, Aβ1–42, t-tau, and p-tau in patients suffering from Parkinson’s disease and SWEDD. Also, we followed the changes in these biomarkers over the course of two years. We observed that MDS-UPDRS, RBD, and UPSIT scores were aggravated in patients suffering from PD and SWEDD when compared to HCs. These results are consistent with previous studies. Moreover, SWEDD patients demonstrated considerably lower MoCA and LNS scores and considerably higher ESS scores in comparison to HCs. MoCA is suggested as a useful screening tool for the early detection of mild cognitive impairment or dementia in PD patients (Hoops et al., 2009). We also observed that the MDS-UPDRS part I and III, UPSIT, and ESS scores significantly differed in SWEDD patients compared to PD patients. Additionally, it can be interpreted from our results that there are significant differences both in motor and non-motor aspects of SWEDD and PD.

Previous studies have shown structural differences in SWEDD and PD patients (Kim and Park, 2016). For instance, in a study investigating the structural differences using diffusion tensor imaging (DTI) in PD patients, SWEDD patients, and normal controls, it was demonstrated that sensorimotor cortex-putamen and pallidum-putamen connections might serve as imaging biomarkers that are unique to SWEDD (Kim and Park, 2016). There are also other studies investigating the structural differences between SWEDD and PD individuals; however, there is limited evidence on the levels of CSF biomarkers in SWEDD subjects and our study is unique in focusing on the difference in the levels of CSF biomarkers between SWEDD and PD individuals.

We observed that the level of α-syn was significantly different between SWEDD and PD individuals after one year and two years but this result is not applicable at 6 months and baseline. Our results indicate that there might be different α-syn pathophysiology in SWEDD patients compared to PD but there is room for more investigations. Previous studies reported that α-syn plays an important role in the pathogenesis of PD (Du, Xie et al., 2020). The exact mechanism by which α-syn contributes to PD is not clearly elucidated yet; however, there are some mechanisms suggested. Some of the proposed mechanisms include mitochondrial dysfunction, endoplasmic reticulum (ER) stress, loss of proteostasis, synaptic impairment, cell apoptosis, and inflammation (Du et al., 2020). In addition, it was supported that blocking α-syn transmission is an important component in the treatment of PD due to the ability of α-syn oligomers to reproduce and propagate themselves (Du et al., 2020). Additionally, the mechanism by which the shift of α-syn occurs is not completely elucidated yet; however, it is proposed that the interaction between the α-syn aggregated in the form of the Lewy body and its secretion resulting from synaptic degeneration can be the responsible mechanism (Parnetti et al., 2019). However, different level of CSF α-syn between PD and SWEDD patients suggests different pathophysiological mechanisms.

Our data demonstrated that the level of Aβ1–42 was significantly different between PD and SWEDD individuals after one year but this result is not applicable for other time points. This result can be another indicator of the different pathophysiology of PD and SWEDD. One of the studies suggested that the source of the different motor and cognition functions between PD and SWEDD is the difference in their white matter tracks. It was ultimately observed that three clusters in the cingulum bundle (CB) were different in PD in comparison with SWEDD and NC versus SWEDD (Feng et al., 2020). Another study suggested that SWEDD patients are more likely to suffer from early cognitive decline compared to individuals suffering from idiopathic PD and the risk of suffering from cognitive decline is greater for them compared to DaTscan-confirmed early-stage PD (Wyman-Chick et al., 2016). These data confirm that there might be different pathophysiology in cognitive decline. Previous studies have suggested that there is a correlation between the metabolism of Aβ1–42 and α-syn (Buddhala et al., 2015). It was suggested that α-syn contributes to protecting the primary cortical neurons from the effects of Aβ1–42 resulting in the decreased rate of caspase-3-mediated cell death (Resende et al., 2013). Based on our findings, patients with SEDD had a higher level of CSF α-syn compared to PD patients. Moreover, it was suggested that α-syn does so via the PI3K/Akt cell survival pathway (Resende et al., 2013). Another possible explanation of the neuroprotective role of α-syn is its direct interaction with Aβ1–42 oligomers (Resende et al., 2013). Additionally, another study supported that the combination of age, sex, Aβ1–42, plasma extracellular vesicles (EVs) α-syn, and plasma EV tau can be useful to identify cognitive dysfunction in individuals suffering from PD (Chung et al., 2021). The combination of Aβ1–42 and α-syn was suggested to be reliable in determining dementia with Lewy body (DLB) (Bibl et al., 2010).

T-tau and p-tau were suggested as potential biomarkers for the detection of cognitive impairments in PD patients along with other CSF biomarkers like Aβ1–42 and α-syn (Chen et al. 2020). Furthermore, we observed that the levels of t-tau and p-tau differed greatly between SWEDD and PD at several time points. However, when comparing the MoCA score as an indicator of cognitive decline between PD and SWEDD there was no significant difference. All these findings suggest that the process of cognitive impairment in patients with SEDD might be different from those with idiopathic PD. Prior studies suggested that tau species may play a role in the progression of cognitive symptoms in PD patients. In addition, genome-wide association studies showed that the gene of tau (MAPT) is related to the risk of PD (Liu et al., 2015). Hyperphosphorylation of tau is the key element in several neurodegenerative diseases including Alzheimer’s disease.

In our study, we found no considerable difference in the levels of CSF biomarkers (α-syn, Aβ1–42, t-tau, and p-tau) between SWEDD individuals and HCs at baseline, 6-month, 1 year, and 2 years while patients with SWEDD had significantly lower cognitive function compared to HCs. This finding might show that the pathophysiology and cognitive decline observed in SWEDD individuals are carried out through other pathways and not the known mechanisms contributing to PD.

During our longitudinal study, we observed that the level of the α-syn in PD patients differs significantly from baseline after one year and two years; however, the same data is not applicable to other groups. The level of Aβ1–42 considerably increased after one year in SWEDD individuals while it showed a decline in PD patients after two years from baseline. The level of p-tau and t-tau increased significantly after 6 months and two years in SWEDD individuals respectively.

Our results suggest different proteinopathies in SWEDD and PD; however, there should be more investigations to confirm our findings including post-mortem studies. We found that there is not a significant difference in the levels of these biomarkers between SWEDD individuals and HCs, while the comparisons for PD and SWEDD were significant at several time points. All these findings suggest that the process of cognitive impairment in patients with SWEDD might be different from those with idiopathic PD. Also, the level of CSF biomarkers increased at some points in SWEDD while the results for PD patients mostly showed a decrease over time. This finding suggests a faster neurodegeneration process in PD patients compared to SWEDD at least on the basis of these biomarkers. Future studies with longer follow-up duration and more sample sizes are necessary to validate our results.

## Ethical approval

Since the data in this paper were obtained from the PPMI database (ppmi.loni.usc.edu), it does not include any research involving human or animal subjects.

## Funding

We do not have any financial support for this study.

## CRediT authorship contribution statement

FN & SK: Designed the study, analyzed the data, and wrote the paper; FN, MN & SK: collected data, analyzed and interpreted the data, and wrote the draft version of the manuscript. The manuscript was revised and approved by all authors.

## Conflict of interest

The authors declare no conflict of interest regarding the publication of this paper.
